# Room-Temperature Direct Polymerization of Glycerol via Ultrasonication

**DOI:** 10.3390/polym18212727

**Published:** 2026-11-03

**Authors:** Yan Irawan, Yenny Meliana, Muhammad Adiguna Rabbuka, Kasbawati Kasbawati, Efri Mardawati, Biqiong Chen, Yeyen Nurhamiyah, Misri Gozan

**Affiliations:** 1 Department of Chemical Engineering, Faculty of Engineering, Universitas Indonesia, Depok 16424, Indonesia; yani004@brin.go.id; 2 Research Center for Chemistry, National Research and Innovation Agency, KST Habibie Serpong, South Tangerang 15314, Indonesia; yenny005@brin.go.id (Y.M.); arabbuka@gmail.com (M.A.R.); 3 Applied Mathematics Laboratory, Department of Mathematics, Universitas Hasanuddin, Kota Makassar 90425, Indonesia; kasbawati@unhas.ac.id; 4 Department of Agro-Industrial Technology, Faculty of Agricultural Industrial Technology, Padjadjaran University, Jatinangor 45363, Indonesia; efri.mardawati@unpad.ac.id; 5 Research Collaboration Center for Biomass and Biorefinery between BRIN and Padjadjaran University, Jatinangor 45363, Indonesia; yeye001@brin.go.id; 6 School of Mechanical and Aerospace Engineering, Queen’s University Belfast, Stranmillis Road, Belfast BT9 5AH, UK; b.chen@qub.ac.uk; 7 Research Center of Biomass and Bioproducts, National Research and Innovation Agency, Jl. Raya Bogor KM 46, Bogor 16911, Indonesia; 8 Research Center for Biomass Valorization, Faculty of Engineering, Universitas Indonesia, Depok 16424, Indonesia

**Keywords:** ultrasonic (USG) technology, glycerol polymerization, hyperbranched, process intensification, degree of polymerization

## Abstract

A study was conducted on the synthesis of glycerol polymerization using ultrasonic (USG) technology. Although ultrasonication has been widely applied in various chemical processes, its role in glycerol polymerization remains insufficiently explored. In this study, polymerization was conducted under initial room-temperature conditions for 150 min using 4 wt% NaOH as a catalyst at an ultrasonic power of 300 W, corresponding to a cumulative energy input of 427,182 J. The process yielded a product with a molecular weight of 697.53 g/mol and a degree of polymerization (DP) of 7.52. FTIR analysis confirmed ether bond formation through characteristic C–O–C stretching bands in the 1033–1210 cm^−1^ region, while the broad O–H band at 3284 cm^−1^ indicated the presence of residual hydroxyl groups consistent with hyperbranched architectures. ^1^H-NMR spectroscopy further verified polymer formation, evidenced by the emergence of a doublet resonance at δ 3.68–3.69 ppm corresponding to ether linkages and the shift of the hydroxyl proton signal from δ 5.11 ppm in glycerol to δ 4.45–4.79 ppm in the polymer. Quantitative analysis using the Fréchet equation yielded a Degree of Branching (DB) of 0.57, characteristic of a well-defined hyperbranched architecture. This high DB ensures a dense periphery of hydroxyl groups, promoting excellent water solubility and versatile functionalization potential.

## 1. Introduction

Polyglycerol is an oligomeric or polymeric ether composed of repeating glycerol units. Depending on the synthetic route, it can form dendritic macromolecules, a class of monodisperse polymers characterized by highly branched, hierarchical architectures [1]. These dendritic structures are categorized into cascades, dendrimers, and hyperbranched polymers. While cascades and dendrimers possess well-defined, symmetrical frameworks, hyperbranched polymers exhibit a more stochastic architecture with variations in branching types and molecular weights [2]. A typical hyperbranched polyglycerol (hPG) comprises three structural motifs: dendritic units (fully substituted), linear units (partially substituted), and terminal units containing unreacted hydroxyl groups.

The synthesis of hPG typically proceeds via base-catalyzed ring-opening polymerization (ROP). This process is driven by nucleophilic attack by alkoxide anions on the epoxide ring of glycidol or the hydroxyl groups of glycerol, yielding a highly branched scaffold with peripheral hydroxyl groups. Previous studies have refined these strategies to achieve architectures that mimic the perfection of dendrimers. For instance, Sunder and Mülhaupt (3000) demonstrated the Ring-Opening Multibranching Polymerization (ROMBP) of glycidol, where the attack of primary or secondary alkoxide anions on the glycidol carbon leads to the formation of hydroxyalkyl ethers [3]. However, traditional synthesis often relies on indirect routes involving epichlorohydrin or glycidol intermediates [4]. These methods suffer from low atom economy, the generation of significant chloride salt by-products, and the requirement for rigorous purification [5,6,7]. Furthermore, epichlorohydrin is highly toxic and carcinogenic [4]. Conversely, direct polymerization of glycerol using homogeneous catalysts (e.g., KOH or NaOH) requires harsh conditions, typically 300–275 °C for 8–24 h under an inert atmosphere [8,9]. While heterogeneous catalysts like calcium oxide (CaO) can lower the required temperature (200–230 °C), they are susceptible to leaching and deactivation due to the water produced during condensation [4]. Computational studies using HyperChem indicate that the base-catalyzed pathway presents a substantial kinetic barrier, reflected in the harsh temperatures and long residence times commonly reported; mechanistic studies highlight the role of in situ formed active glycerolates and S_N_2-type etherification steps [2], highlighting the reaction’s significant energy barrier.

Ultrasonic technology (USG) has emerged as a powerful tool for process intensification in polymer chemistry. Beyond its established role in the controlled depolymerization of macromolecules [10,11], ultrasonication significantly enhances reaction kinetics through acoustic cavitation. The formidable energy barrier of 529.38 KJ/mol for glycerol etherification typically necessitates harsh thermal conditions. This study demonstrates that acoustic cavitation effectively bypasses this requirement through the ‘hotspot’ mechanism. The rapid collapse of cavitational bubbles generates localized temperatures of 5000 K and pressures of 1000 atm, providing the necessary activation energy at a molecular scale while maintaining the bulk temperature near ambient. The inherent high viscosity of glycerol often constrains reactant mass transfer. However, acoustic cavitation induces micro-jets and acoustic streaming at velocities up to 100 m/s. This promotes the interfacial collision frequency between glycerolate anions and glycerol molecules, ensuring the effective utilization of hotspot-generated energy to drive the polymerization kinetics. In biodiesel synthesis, for example, ultrasonic horn systems achieved a 93% conversion at 45 °C in just 45 min, significantly outperforming conventional methods (39%) under identical conditions [12].

Previous studies on glycerol polymerization have predominantly focused on indirect routes involving glycidol or epichlorohydrin intermediates, which often suffer from low atom efficiency, complex purification steps, and environmental concerns related to chlorinated by-products. Direct glycerol polymerization using alkaline catalysts has been proposed as an alternative; however, reported processes typically require temperatures above 300 °C and reaction times exceeding several hours.

Despite the advantages of sonochemistry, the feasibility of synthesizing dendritic polyglycerol via direct polymerization under mild conditions remains insufficiently explored. Most literature focuses on high-temperature indirect routes, leaving a gap in the systematic understanding of how ultrasonication facilitates ether bond formation at lower temperatures and shorter reaction times.

This study investigates the efficacy of ultrasonication as a process intensification tool for the direct polymerization of glycerol. We aim to elucidate the influence of ultrasonic power and energy input on the resulting molecular weight, Degree of Branching, and the formation of dendritic architectures under mild conditions. By correlating sonochemical parameters with the structural and physicochemical properties of the polyglycerol, this work seeks to provide mechanistic insights into ultrasound-assisted polymerization and evaluate its potential as a sustainable, low-energy alternative to conventional thermal processes.

## 2. Materials and Methods

### 2.1. Material

The materials used in the synthesis of PG using sonification are technical-grade glycerol 96% (PT. Brataco, Jakarta, Indonesia), Analytic-grade potassium hydroxide ≥ 85% (Merck, Darmstadt, Germany), Analytic-grade sodium hydroxide ≥ 99% (Merck) and cation-exchange resin (Lewatit S-1467, Lanxess, Cologne, Germany).

### 2.2. Synthesis Process

The PG synthesis process using USG technology employed an Ultrasonic Horn unit, model VCX750/VCX500, from Sonic & Material, Inc. (Newtown, CT, USA). The experimental setup of the apparatus is illustrated in Figure 1. The method used in this activity is direct polymerization, in which glycerol is polymerized using strong, homogeneous base catalysts such as KOH or NaOH. In this activity, several process variables affecting the formation of glycerol monomers were investigated, including the molecular weight and chemical structure of the resulting PG. Process variables included KOH and NaOH catalysts (4 wt%), ambient temperature, a frequency of 20 kHz, and ultrasonic power levels of 100, 150, and 300 W (corresponding to 20, 30, and 40% amplitudes). The process begins by reacting 98% glycerol with a 4% KOH/NaOH catalyst by weight of 98% glycerol for 150 min. After the process, purification is performed to separate the KOH/NaOH catalyst from the product using absorption techniques with cation exchange resin (Lewatit S-1467). After achieving a neutral pH of 6–7, the product is characterized using FTIR, NMR, and molecular weight analysis via the Ostwald viscometer method.

### 2.3. Identification and Characterization

The PG product’s identification and characterization included determining functional groups. Identification was performed using a Fourier Transform Infrared Spectrophotometer (IRPrestige-21, Shimadzu, Kyoto, Japan); chemical structure analysis was conducted using Nuclear Magnetic Resonance Spectroscopy (AVANCE III HD 700 MHz, Bruker, Billerica, MA, USA), and molecular weight was determined using the Ostwald viscometry method (Mark–Houwink).

### 2.4. Determination of Viscosity of Glycerol and Polyglicerol (PG)

#### 2.4.1. Determination of Glycerol Viscosity

The viscosity of glycerol solutions with concentrations of 10%, 20%, 30%, 50%, 70%, and 100% was measured using an Ostwald viscometer. The time required for the sample to flow from the upper limit to the lower limit of the viscometer was recorded. Measurements were taken three times.

#### 2.4.2. Determination of PG Viscosity

The viscosity of PG solutions with concentrations of 10%, 20%, 30%, 50%, 70%, and 100% was measured using an Ostwald viscometer. The time required for the sample to flow from the upper limit to the lower limit of the viscometer was recorded. Measurements were taken three times.

### 2.5. Determination of Density/Mass Density of Glycerol and Polyglycerol (PG)

Density measures the amount of substance contained in a unit volume. The density of the solution was measured using a 25 mL pycnometer. The solution was made by mixing glycerol with water and PG with water at various concentrations. The formula for determining density is:

(1) $$\rho =\frac{m}{v}$$

where ρ *represents* the density (g/mL), m is the mass of the sample (g), and v is the volume of the sample (mL).

#### 2.5.1. Determination of Glycerol Density

Weigh the empty pycnometer and record its mass. Insert the glycerol solution samples with concentrations of 10%, 20%, 30%, 50%, 70%, and 100% up to the neck of the pycnometer, weigh the filled pycnometer, and then record and calculate the density.

#### 2.5.2. Determination of PG Density

Weigh the empty pycnometer and record its mass. Insert the PG solution sample with concentrations of 10%, 20%, 30%, 50%, 70%, and 100% up to the neck of the pycnometer, weigh the filled pycnometer, and then record and calculate the density.

##### Determination of Intrinsic Viscosity Using the Least Squares Method

The intrinsic viscosity [η] was determined using the Least Squares method by calculating the relative viscosity [η_rel_], specific viscosity [η_sp_], and reduced viscosity [ηred]. Relative viscosity was determined by the ratio between the sample solution flow time (t) and the pure solvent flow time (t0). Specific viscosity (η_sp_) and reduced viscosity (η_red_) were determined using the following equations [13]:

(2) $$\mathrm{Relative}\;\mathrm{viscosity}:\;\eta_{rel}=\frac{t}{t_{0}}$$

(3) $$\mathrm{Specific}\;\mathrm{viscosity}:\;\eta_{sp}=\eta_{rel}-1$$

(4) $$\mathrm{Reduced}\;\mathrm{viscosity}:\;\eta_{red}=\frac{\eta_{sp}}{C}$$

##### Determination of Molecular Weight Using the Mark–Houwink Equation

The viscosity-average molecular weight (M_v_) of the synthesized polyglycerol products was determined via the intrinsic viscosity method using the Mark–Houwink–Sakurada (MHS) equation, rather than conventional size-exclusion chromatography (GPC/SEC). This physical approach was selected because polyglycerol typically exhibits a hyperbranched architecture, wherein linear GPC calibration standards tend to underestimate the actual molecular weight due to discrepancies in hydrodynamic volume ([η] α V_h_/M) [14,15,16]. Furthermore, intrinsic viscosity directly assesses the hydrodynamic dimensions and solute–solvent interactions ([η] α V_h_/M) without the risk of peak distortion caused by non-specific adsorption of the highly polar polymer onto the aqueous GPC stationary phase. Consequently, this method offers superior physical sensitivity for accurately monitoring conformational changes induced by the variations in ultrasonication conditions and base catalysts (KOH vs. NaOH).

To experimentally determine the intrinsic viscosity (η), the polyglycerol samples were first dried in a vacuum oven at 60 °C for 24 h to ensure complete moisture removal. The dehydrated samples were then dissolved in deionized water to prepare a series of solutions with varying concentrations (c). Viscosity measurements were subsequently performed using a Ubbelohde capillary viscometer immersed in a thermostatic water bath maintained at a constant temperature of 25 °C.

From the experimental flow times, the reduced viscosity and inherent viscosity were calculated. The intrinsic viscosity (η) was then determined through the least-squares method by extrapolating both the Huggins and Kraemer plots to zero concentration. Finally, the obtained [η] value was applied to calculate the molecular weight using the empirical MHS relationship, as expressed in Equation (5):

(5) $$\left[\eta \right]=KM^{\alpha }$$

where [η] is the intrinsic viscosity, Mv represents the viscosity-average molecular weight, while K and α are the characteristic Mark–Houwink constants for the specific polymer-solvent system. For the polyglycerol-water system at 25 °C, the constants were defined as K = 2 × 10^−5^ L/g and alpha = 0.71 [17], in accordance with the established literature.

##### Determination of Degree of Polymerization (DP)

The degree of polymerization (DPn) was determined by measuring the intrinsic viscosity (η) using an Ubbelohde viscometer at 25 °C, which was subsequently converted using the Mark–Houwink–Sakurada equation (Equation (5)) to obtain the viscosity-average molecular weight (Mv).

(6) $${DP}_{n}=\frac{M_{v}-M_{H_{2}O}}{M_{0}}=\frac{M_{v}-M_{H_{2}O}}{\left(M_{Glycerol}-M_{H_{2}O}\right)}$$

where:

Molecular Weight Glycerol (M glycerol) = 92.09 g/mol

Molecular Weight Water (M H_2_O) = 18.02 g/mol

Repeat Unit Molecular Weight (M_0_) = 92.09–18.02 = 74.07 g/mol

##### Determination of Degree of Branching (DB)

The Degree of Branching (DB) was calculated based on the ^1^H-NMR signal intensity ratios of the dendritic (D), terminal (T), and linear (L) units within the polymer framework. For polyglycerol (PG), the DB is calculated based on the relative integration of the dendritic (D), linear (L), and terminal (T) structural units identified in the spectrum, using the Frey equation [18,19]:

(7) $$DB=\frac{D+T}{D+L+T}$$

where ***D***, ***L*** and ***T*** represent the integral areas of the dendritic, linear and terminal.

## 3. Results

The PG synthesis process utilizes ultrasonic technology (USG) through an ultrasonic horn unit. This process involves direct polymerization, in which the NaOH/KOH base catalyst interacts with glycerol, weakening one of its OH bonds and enhancing the nucleophilic character of the hydroxyl oxygen. Subsequently, glycerol rapidly attacks the carbon atom of a second glycerol molecule, forming an ether bond and producing diglycerol, with water released.

### 3.1. Effect of USG on Process Temperature Increase

To evaluate the role of ultrasonication as a process intensification tool, it is essential to monitor the temperature evolution during polymerization. Although the reaction was initiated at room temperature, ultrasonic energy input may induce bulk heating due to acoustic energy dissipation and cavitation phenomena within the reaction medium. Therefore, the temperature was continuously recorded over 150 min at ultrasonic powers of 100, 150, and 300 W using 4% KOH and 4% NaOH as catalysts.

The resulting temperature profiles are presented in Figure 2 to assess the influence of ultrasonic power on the system’s thermal behavior during glycerol polymerization.

As shown in Figure 2a,b, all power levels exhibit a rapid increase in temperature during the first 30 min of reaction, followed by a stabilization phase that extends to 150 min. For the KOH-catalyzed system, the maximum temperature reached 86 °C at 300 W. In contrast, the NaOH-catalyzed system reached a higher maximum temperature of 96 °C at the same ultrasonic power. At lower power levels (100 and 150 W), the final temperatures were correspondingly lower for both catalysts. Overall, increasing ultrasonic power led to higher bulk temperatures throughout the reaction at 300 W.

The observed temperature rise indicates that, although the reaction was initiated at room temperature, the system does not remain thermally neutral during ultrasonication. The applied acoustic energy is partially converted into thermal energy through cavitation-induced microbubble collapse and viscous dissipation. At an ultrasonic frequency of 20 kHz, each collapsing cavitation bubble can generate localized “hotspots” with transient temperatures of approximately 5000 °C and pressures up to 3000 atmospheres [20,21]. While these extreme conditions are spatially and temporally confined, their cumulative effect contributes to the measurable increase in bulk temperature.

Importantly, even at the highest ultrasonic power (300 W), the bulk temperature remained below 100 °C (86–96 °C), which is significantly lower than those in conventional glycerol polymerization processes, which typically require temperatures above 300 °C. This suggests that the enhanced polymerization observed under ultrasonication cannot be attributed solely to macroscopic heating, but also to sonochemical activation effects at the microscale.

### 3.2. Effect of USG on Process Energy Increase

In addition to temperature evolution, the cumulative energy input during ultrasonication is critical for determining reaction progress and polymer growth. Since ultrasonic power directly governs acoustic intensity, it is necessary to quantify the total energy delivered to the reaction system over time.

Therefore, the cumulative energy supplied during 150 min of polymerization was calculated for ultrasonic powers of 100, 150, and 300 W using 4% KOH and 4% NaOH as catalysts. The energy–time profiles are presented in Figure 3 to evaluate how ultrasonic power influences total process energy input.

As shown in Figure 3a,b, cumulative energy increases progressively with reaction time for all power levels. Higher ultrasonic power results in a steeper slope of the energy–time curve, indicating a greater rate of energy delivery. For the KOH-catalyzed system, the maximum cumulative energy reached 360,593 J at 300 W after 150 min. In the NaOH-catalyzed system, the cumulative energy reached 427,182 J at the same ultrasonic power and reaction time. In Figure 3b, a noticeable increase in slope is observed between 60 and 70 min at 300 W, suggesting a change in energy accumulation behavior under NaOH catalysis. Overall, increasing ultrasonic power results in higher cumulative energy input over the reaction duration for both catalytic systems.

The linear increase in cumulative energy with time reflects continuous acoustic energy transfer from the ultrasonic horn into the reaction medium. As ultrasonic power increases, the intensity and frequency of cavitation events increase accordingly, leading to greater energy dissipation within the liquid phase.

At an ultrasonic frequency of 20 kHz, collapsing cavitation bubbles generate localized transient hotspots with temperatures of approximately 5000 K and pressures exceeding 1000 atm [22,23]. Although these extreme conditions occur on the microscale, their repetitive occurrence results in substantial cumulative energy input at the macroscopic level. The higher cumulative energy observed in the NaOH system (427,182 J) compared to the KOH system (360,593 J) at identical power and time suggests differences in energy transfer efficiency and/or system response under different catalytic environments. This may influence reaction kinetics, molecular weight development, and the formation of branching structures.

Importantly, the cumulative energy input provides a more representative parameter for process intensification analysis than nominal power alone. While power defines the instantaneous energy-delivery rate, cumulative energy reflects the total acoustic work imparted to the system, which is directly relevant to polymer growth and structural evolution.

### 3.3. Effect of USG on Molecular Weight (MW) and Degree of Polymerization (DP) Increase

Beyond temperature and cumulative energy input, the most critical indicators of polymerization performance are molecular weight (MW) and degree of polymerization (DP). Since polymer growth often manifests initially as macroscopic physical changes, such as increased viscosity and color variation, both qualitative visual observation and quantitative molecular characterization are necessary to evaluate the effect of ultrasonic power. Therefore, visual inspection of the reaction products (Figure 4) was first performed to identify physical changes associated with increasing ultrasonic intensity. These observations were then correlated with viscosity-based molecular weight determination and DP calculation using the Mark–Houwink relationship, as summarized in Table 1.

As shown in Figure 4, raw glycerol appears colorless and transparent. With increasing ultrasonic power (100, 150, and 300 W), the resulting PG samples exhibit progressively darker coloration for both catalytic systems. For the KOH system (Figure 4a), the product transitions from nearly colorless at 100 W to light yellow at 300 W. In the NaOH system (Figure 4b), the color change is more pronounced, reaching a deeper yellow tone at 300 W compared to the corresponding KOH sample. These visual changes suggest progressive modification of the reaction mixture as ultrasonic intensity increases.

Table 1 shows that viscosity increases with increasing ultrasonic power for both catalytic systems. For the NaOH-catalyzed samples, viscosity increased from 0.671 (100 W) to 2.089 (300 W). Similarly, in the KOH system, viscosity increased from 0.705 (100 W) to 1.617 (300 W). Correspondingly, molecular weight increased with ultrasonic power. The highest MW was obtained for the HPG.N4.40 sample (NaOH, 300 W), with a MW of 697.53 g/mol and a DP of 7.52, confirming a successful transition into the polymeric regime (DP > 5). In contrast, the highest MW in the KOH system (HPG.K4.40, 300 W) reached 482.04 g/mol, with a DP of 5.20. At lower ultrasonic power levels (100–150 W), the low MW and DP values (ranging from 1.52 to 3.41) indicate that the products reside strictly within the oligomeric regime, consisting predominantly of dimers and trimers. Across all conditions, the NaOH system consistently yielded higher viscosity, molecular weight, and DP compared to the KOH system at the same ultrasonic power.

The progressive increase in viscosity, MW, and DP with ultrasonic power indicates enhanced chain growth and structural development under higher acoustic energy input. Since intrinsic viscosity is directly related to hydrodynamic volume and molecular chain length via the Mark–Houwink relationship, the observed increase in viscosity confirms the formation of longer molecular chains as the reaction progresses from oligomeric species into more distinct polymeric architectures at 300 W. The stronger color development at higher power levels, particularly in the NaOH system, may be associated with increased molecular complexity and possible side reactions occurring under intensified cavitation conditions. The correlation between darker coloration and higher MW suggests that ultrasonic intensity influences not only chain extension but also structural architecture. Importantly, the NaOH catalyst consistently produced higher MW and DP than KOH under identical ultrasonic conditions. This may reflect differences in base strength, ionic mobility, or glycerolate formation efficiency, which could influence nucleophilic activation and ether bond formation during the condensation process.

When considered alongside Section 3.1 and Section 3.2, these results suggest that cumulative acoustic energy, rather than nominal temperature alone, plays a decisive role in determining oligomer and polymer growth. The enhancement in MW at bulk temperatures below 100 °C further supports the contribution of localized sonochemical activation beyond conventional thermal effects.

### 3.4. Characterization of PG Using FTIR

Fourier Transform Infrared (FTIR) spectroscopy was performed to confirm the successful polymerization of glycerol into polyglycerol (PG) and to identify the characteristic functional groups. This analysis allows for the tracking of chemical bond transformations, specifically the formation of ether linkages and the retention of hydroxyl groups. The comparative FTIR spectra of monomeric glycerol and the synthesized PG are displayed in Figure 5, with the detailed assignment of absorption bands summarized in Table 2.

As illustrated in Figure 5 and Table 2, both spectra exhibit a broad and intense absorption band centered at 3285.61 cm^−1^, which is explicitly assigned to the stretching vibrations of hydroxyl (O–H) groups. This prominent band indicates a dense network of hydrogen bonding among the polyol units. The retention of this intense hydroxyl signal in the PG spectrum suggests that a significant number of terminal hydroxyl groups remain intact, a characteristic feature that aligns well with the formation of a hyperbranched polymer architecture [24]. Furthermore, the sharp absorption doublets observed at 2925.84 cm^−1^ and 2873.17 cm^−1^ correspond to the asymmetric and symmetric stretching vibrations of aliphatic C–H (CH)_2_ groups, confirming the preservation of the aliphatic backbone after polymerization.

The definitive evidence of successful glycerol intermolecular condensation is demonstrated by the evolution of the fingerprint region. The strong, complex absorption band at 1031.85 cm^−1^ represents the stretching vibrations of ether (C–O–C) linkages overlapping with residual primary and secondary alcohol (C–O) bonds [25]. This multi-peak profile in the 1415.10–1100 cm^−1^ region—associated with coupled C–H bending and C–O stretching—further supports the formation of a polyether framework.

Interestingly, a distinct peak appears at 1655.85 cm^−1^, which can be attributed to either localized C=O carbonyl stretching from minor oxidative side reactions or tightly bound/absorbed water molecules (O–H bending) within the hygroscopic polymer matrix [26]. The simultaneous coexistence of robust ether (C–O–C) bonds and abundant residual C–O\O–H groups underscores that the product is a partially substituted, highly branched macromolecule rather than a fully cross-linked or linear structure.

In conclusion, the FTIR analysis confirms that ultrasonic irradiation efficiently drives the etherification of glycerol into a polyether network while strategically preserving terminal hydroxyl functionalities essential for subsequent dendritic functionalization [27].

### 3.5. Characterization of PG Using ^1^H-NMR

While FTIR confirms the presence of ether and hydroxyl functional groups, ^1^H-NMR spectroscopy provides more detailed structural insight into proton environments and enables differentiation between glycerol and its polymerized form. In particular, ^1^H-NMR allows identification of newly formed ether linkages and changes in hydroxyl proton behavior, which are critical indicators of successful polymerization.

To evaluate structural evolution at the highest molecular weight condition, the ^1^H-NMR spectrum of glycerol was compared with that of PG.N4.40 (NaOH catalyst, 300 W, 150 min), as shown in Figure 6.

The structural transition from monomeric glycerol to polyglycerol (PGL) was unambiguously monitored via ^1^H-NMR spectroscopy. As shown in Figure 6a, pristine glycerol displayed well-defined multiplets in the range of δ = 3.65–3.85 ppm corresponding to the aliphatic backbone, along with a single proton exchange peak for hydroxyl groups at δ = 5.11 ppm. In contrast, the PGL spectrum (Figure 1) in DMSO-d_6_ exhibited a distinct fingerprint of a highly branched macromolecular architecture. The emergence of a predominant, sharp resonance at δ = 3.69 ppm, assigned to the CH2–O–CH2 linkages, provides definitive evidence for successful etherification and backbone propagation. Furthermore, taking advantage of the suppressed proton exchange in DMSO-d_6_, the hydroxyl region was resolved into three distinct signals at δ = 4.56, 4.63 and 4.79 ppm. These triplets are highly characteristic of the coexistence of dendritic, linear, and terminal structural units, verifying the hyperbranched nature of the synthesized polymer.

### 3.6. Determination of the Degree of Branching (DB) of Polyglycerol

Based on the integration values shown in the ^1^H-NMR spectrum (Figure 7), the structural units and the Degree of Branching (DB) were estimated. The Degree of Branching (DB) of the synthesized hyperbranched polyglycerol was determined quantitatively from the ^1^H-NMR spectrum (in DMSO-d_6_) using the Frey equation based on the integral areas of the hydroxyl (-OH) protons. The structural units of polyglycerol consist of dendritic (D), linear (L), and terminal (T) units, where the stoichiometric balance dictates that D ≈ T in a randomly branched step-growth system.

As illustrated in Figure 7, the resonances in the 4.56–4.62 ppm region are assigned to hydroxyl protons (-OH), while the 3.30–3.66 ppm range represents a superposition of methylene (-CH_2_-) and methine (-CH-) protons from the dendritic (D), linear (L), and terminal (T) backbone units. Peaks D, E, and F (3.43–3.35 ppm) are assigned to linear and terminal units (integrations: 0.88, 1.03, 0.92), with peak G (3.30 ppm) providing essential terminal (T) unit information to calculate the Degree of Branching (DB) via the Frey equation.

Based on the structural unit quantification data in Table 3, the degree of branching (DB) value was determined using the following equation:

$$DB=\frac{D+T}{D+L+T}=\frac{2.44+4.91}{2.44+5.63+4.91}=0.566=0.57$$

The structural architecture of the glycerol polymer synthesized via ultrasonication (USG) was quantitatively characterized using ^1^H-NMR spectroscopy. Based on the Frey equation and the integral areas of the dendritic (D), linear (L_1,2_, L_1,3_), and terminal (T) units, the Degree of Branching (DB) was calculated to be 0.57. This value aligns closely with the theoretical DB of 0.50 typically associated with the slow monomer addition (SMA) technique in Ring-Opening Multibranching Polymerization. A DB of 0.57 signifies a well-defined hyperbranched topology, reflecting a statistical distribution of branching points intermediate between perfectly linear (DB = 0) and perfectly dendritic (DB = 1) structures. This architectural configuration yields a high density of peripheral hydroxyl groups, which is essential for subsequent functionalization and superior aqueous solubility.

## 4. Discussion

The results demonstrate that ultrasonication (USG) effectively intensifies glycerol polymerization. Upgrading surplus crude glycerol into high-value hyperbranched polyglycerol aligns with recent advancements in process intensification and reaction engineering for green biorefineries [28,29]. Under ultrasonic conditions, polymerization proceeds from an initial room-temperature state and reaches significant molecular growth within 150 min. Structural evidence from ^1^H-NMR analysis (Figure 6 and Figure 7) confirms the formation of a branched polyglycerol architecture, indicating that ultrasonication promotes not only chain extension but also branching development. The reaction pathway follows the base-catalyzed glycerol polymerization mechanism described by Martin and Richter, involving sequential nucleophilic substitution and ether bond formation. The proposed stages of linear and branched polymer growth are illustrated in Figure 8.

Quantitatively, the NaOH-catalyzed system achieved higher molecular weight and degree of polymerization than the KOH system at each equivalent operational amplitude level. At the highest amplitude (40%), the NaOH-catalyzed sample (HPG.N4.40) achieved a molecular weight of 697.53 g/mol with a degree of polymerization (DP = 7.52), whereas the KOH-catalyzed sample (HPG.K4.40) yielded only 482.04 g/mol with a DP of 5.20. This phenomenon correlates directly with the efficiency of mechanical energy transfer from the ultrasonic vibrations into the 250 mL reaction medium, wherein the NaOH system exhibits significantly more progressive molecular growth kinetics and product viscosity enhancement (2.089 mPa·s for NaOH vs. 1.607 mPa·s for KOH).

The superior performance of the NaOH-catalyzed system over KOH under ultrasonic activation can be explained through differences in cation charge density and ion-pairing effects. The smaller ionic radius of Na^+^ (0.95 Å) compared to K^+^ (1.33 Å) imparts a higher charge density to the sodium cation [30,31]. This higher charge density enhances the polarization of the glycerol hydroxyl groups, facilitating proton abstraction to form highly reactive glycerolate alkoxide nucleophiles. Furthermore, under the extreme transient conditions generated by acoustic cavitation, the tighter ion-pairing of Na^+^-glycerolate provides a more optimal spatial orientation for the subsequent nucleophilic attack stages [32,33].

Ultrasonication generates alternating compression and rarefaction cycles in the liquid medium, leading to the formation, growth, and implosive collapse of cavitation bubbles. The collapse of these bubbles produces localized transient hotspots with extreme temperatures (5000 °C) and pressures (3000 atm), as reported in the literature. Although these extreme conditions are confined to microdomains, their repeated occurrence facilitates enhanced molecular-level reactant activation and bond rearrangement. In addition to localized thermal effects, cavitation-induced microturbulence enhances mixing efficiency and mass transfer between glycerol molecules and the base catalyst. This increases the likelihood of nucleophilic substitution, accelerating ether bond formation and reducing the effective time required for polymer growth.

Compared with conventional glycerol polymerization processes, which typically require temperatures above 300 °C and extended reaction times, the ultrasonic-assisted system achieves polymer growth at substantially lower bulk temperatures. Table 4 summarizes representative conventional polymerization conditions reported in the literature [4], highlighting the contrast in operational severity.

The base-catalyzed pathway presents a substantial kinetic barrier, reflected in the harsh temperatures and long residence times commonly reported; mechanistic studies highlight the role of in situ formed active glycerolates and S_N_2-type etherification steps. This high interaction energy suggests the reaction intrinsically involves a significant energetic barrier. The ability of ultrasonication to facilitate polymerization under mild bulk conditions therefore supports the hypothesis that localized cavitation energy contributes to transient activation states that lower the effective kinetic barrier.

Taken together, the combined thermal, mechanical, and microreactor effects generated by ultrasonication explain the observed increases in molecular weight, degree of polymerization, and branching. The results suggest that cumulative acoustic energy, rather than bulk temperature alone, is the dominant factor governing polymer growth and structural development in this system.

## 5. Conclusions

This study demonstrated glycerol polymer synthesis via ultrasonic-assisted polymerization as a highly efficient process intensification strategy. By harnessing acoustic cavitation, the reaction effectively bypassed the substantial kinetic barrier of 529.38 kJ/mol under near-ambient bulk temperatures, a significant departure from the extreme thermal requirements of conventional methods.

Characterization results confirmed the formation of a hyperbranched architecture with a molecular weight of 697.53 g/mol and a Degree of Branching (DB) of 0.57, as verified by FTIR and NMR spectroscopy. FTIR analysis confirmed ether bond formation through characteristic C–O–C stretching bands in the 1033–1210 cm^−1^ region, while the broad O–H band around 3284 cm^−1^ indicated the presence of residual hydroxyl groups consistent with hyperbranched architectures. ^1^H-NMR analysis further validated polymer formation through the emergence of ether-related resonances and the shift and broadening of hydroxyl proton signals, reflecting structural evolution from monomeric glycerol to a branched polymer framework. The calculated DB of 0.57 confirms the successful synthesis of hyperbranched polyglycerol via ultrasonication. This specific architectural configuration—intermediate between linear and perfectly dendritic structures—is instrumental in achieving superior aqueous solubility and providing a robust platform for subsequent chemical modifications.

Increasing ultrasonic power led to systematic increases in viscosity, molecular weight, and DP, indicating that cumulative acoustic energy plays a decisive role in polymer growth. The NaOH catalyst consistently produced higher molecular weights and branching levels than KOH under identical ultrasonic conditions, suggesting more efficient nucleophilic activation and glycerolate formation.

Compared to conventional glycerol polymerization processes that typically require temperatures above 300 °C and prolonged reaction times, the ultrasonic-assisted system enables polymer growth at bulk temperatures below 100 °C. The results support the hypothesis that localized cavitation effects enhance reaction kinetics beyond simple thermal heating. Despite its promising performance, further work is required to evaluate scale-up feasibility, optimize energy efficiency, and assess reactor design considerations for continuous-flow ultrasonication. Future research should also explore detailed kinetic modeling and life-cycle energy analysis to fully quantify the advantages of ultrasonic intensification. In addition, the hyperbranched polyglycerol produced via this method may be further investigated for applications in biomedical, pharmaceutical, or surfactant systems where branching density and hydroxyl functionality are critical.

## Figures and Tables

**Figure 1:**
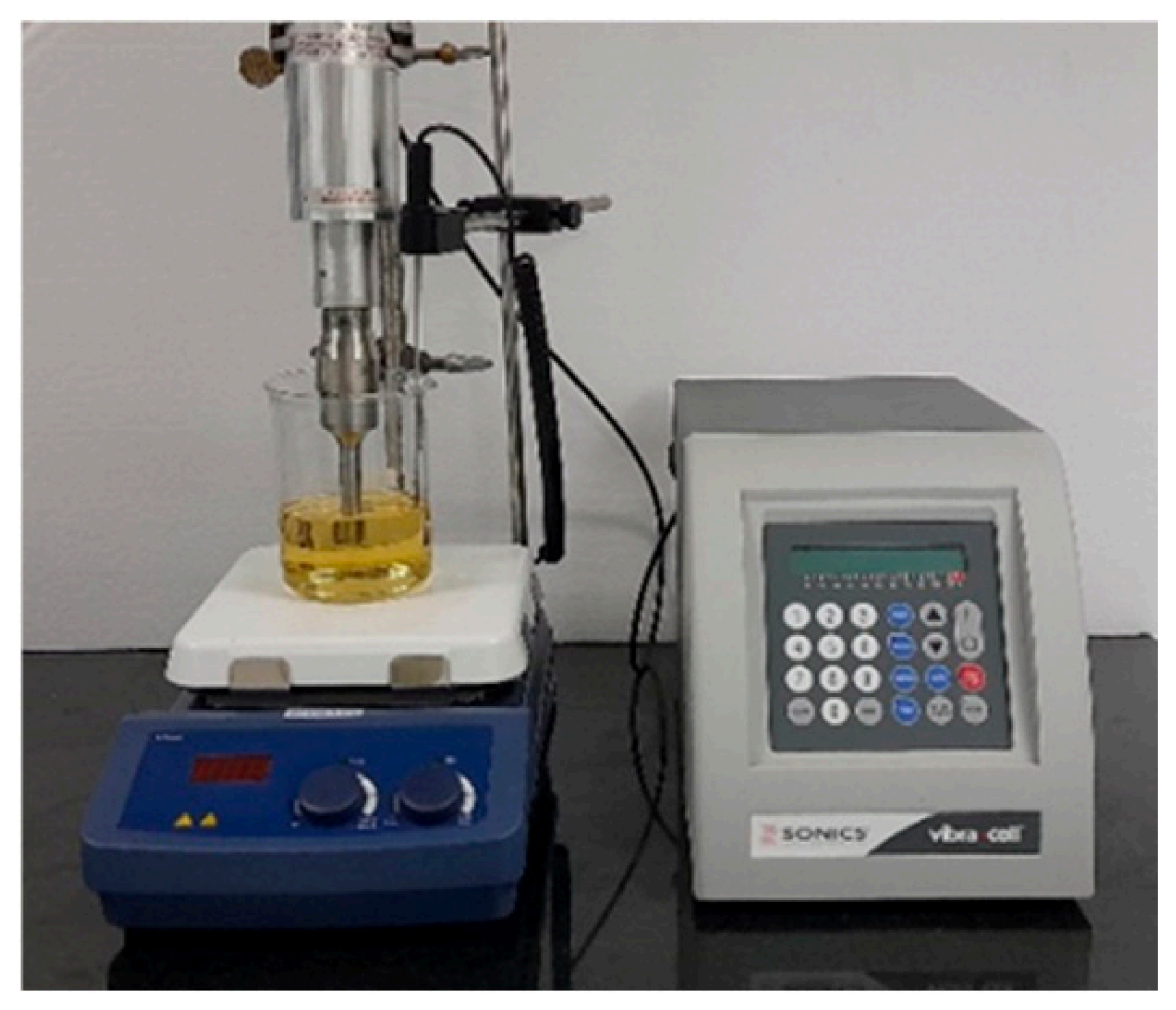
Ultrasonic horn.

**Figure 2:**
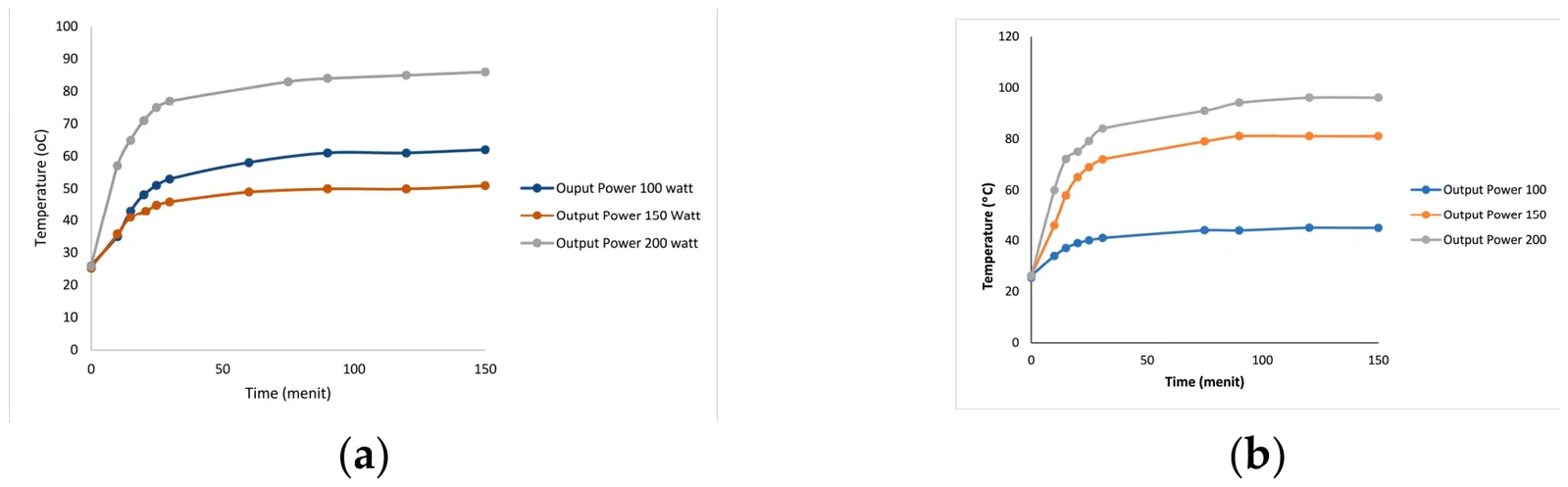
The effect of ultrasonic power on temperature increases polymerization with (**a**) KOH and (**b**) NaOH catalysts.

**Figure 3:**
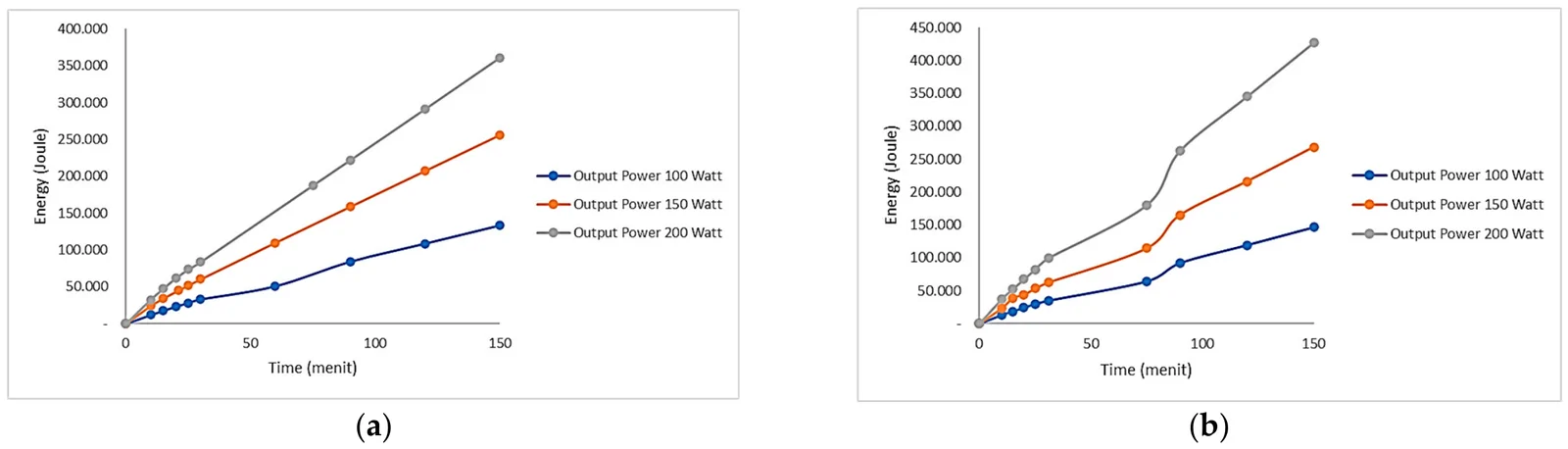
Effect of ultrasonic power on cumulative energy input during glycerol polymerization using (**a**) KOH and (**b**) NaOH catalysts.

**Figure 4:**
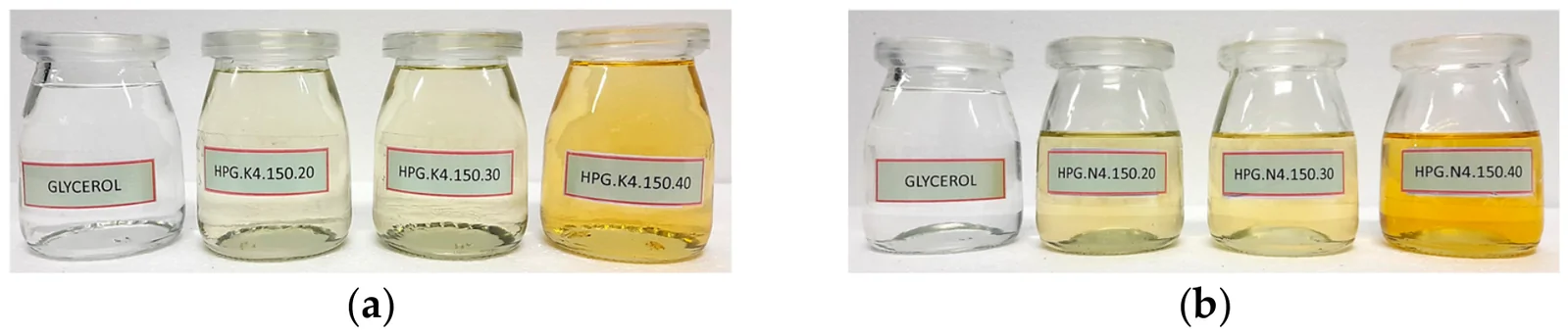
(**a**) Glycerol raw material, PG.K4.150.20: PG product catalyst KOH, polymerization 150 min, ultrasound dose 100 watts, PG.K4.150.30: PG product catalyst KOH, polymerization 150 min, ultrasound dose 150 watts, PG.K4.150.40: PG product catalyst KOH, time 150 min ultrasound dose 300 watts; (**b**) Glycerol raw material, PG.N4.150.20: PG product catalyst NaOH, polymerization 150 min, ultrasound dose 100 watts, PG.N4.150.30: PG product catalyst NaOH, polymerization 150 min, ultrasound dose 150 watts, PG.N4.150.40: PG product catalyst NaOH, polymerization 150 min, ultrasound dose 300 watts.

**Figure 5:**
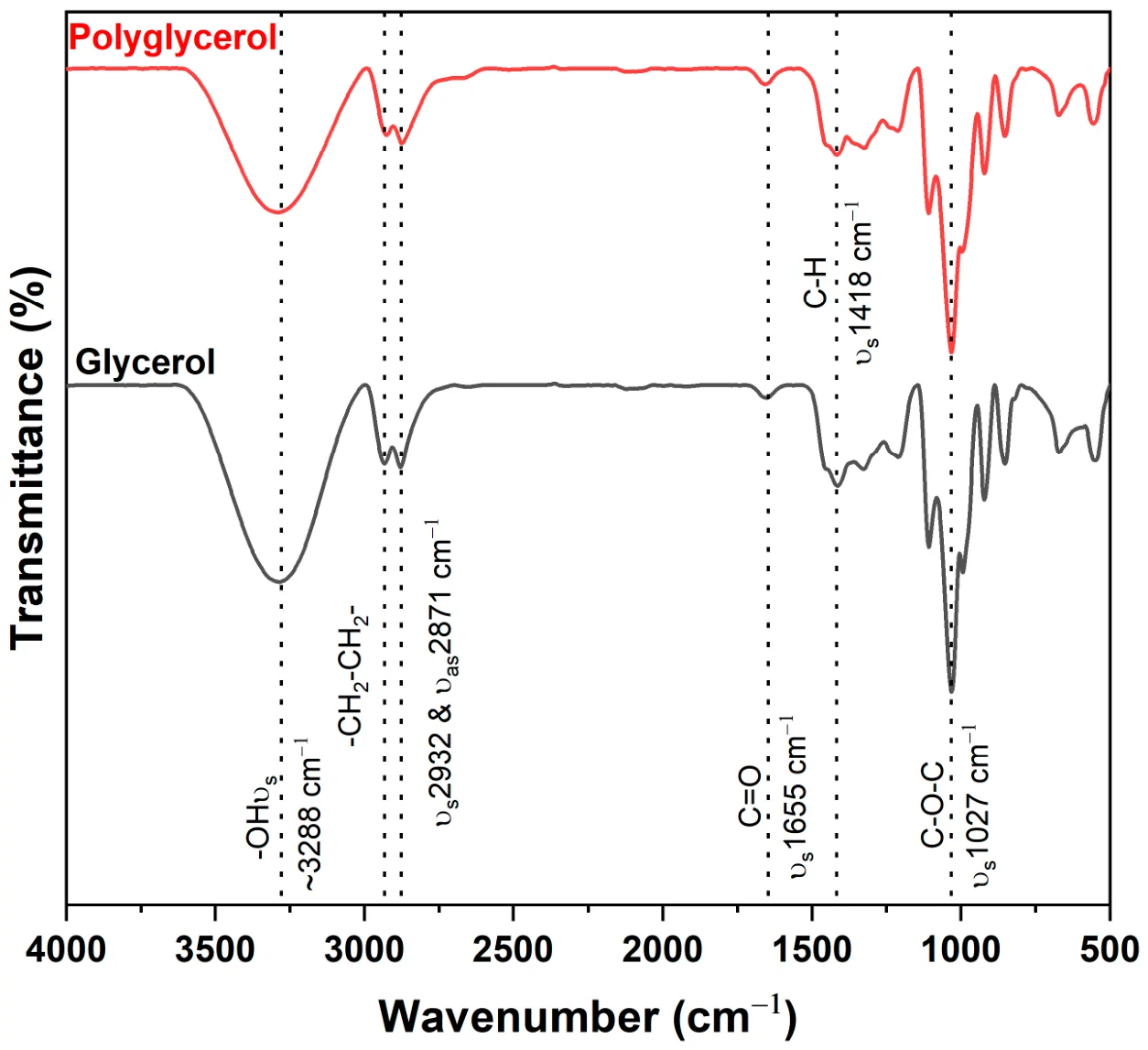
FTIR spectrum of polyglycerol.

**Figure 6:**
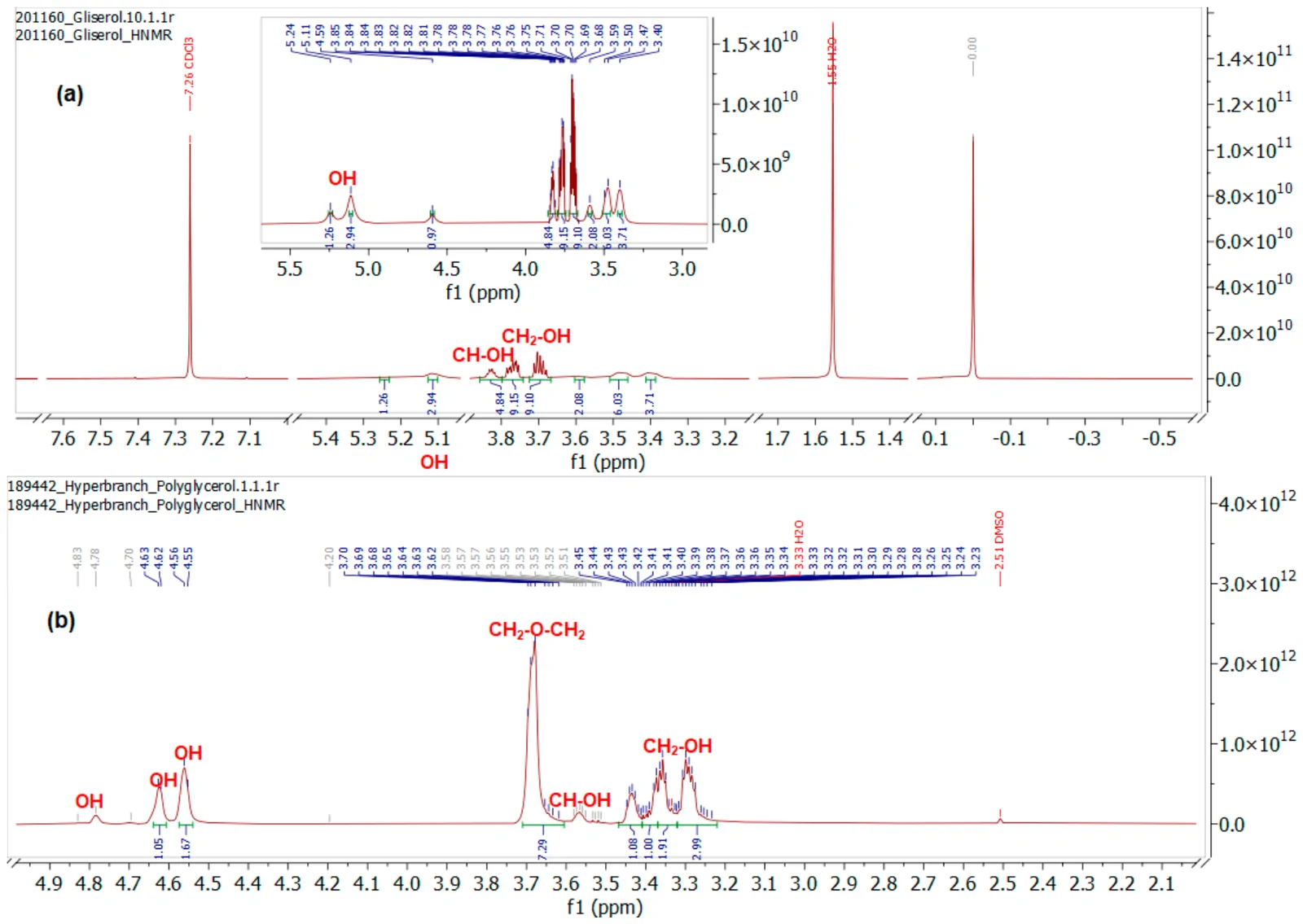
^1^H NMR spectrum of (**a**) glycerol and (**b**) PG.N4.40 at time 150 min.

**Figure 7:**
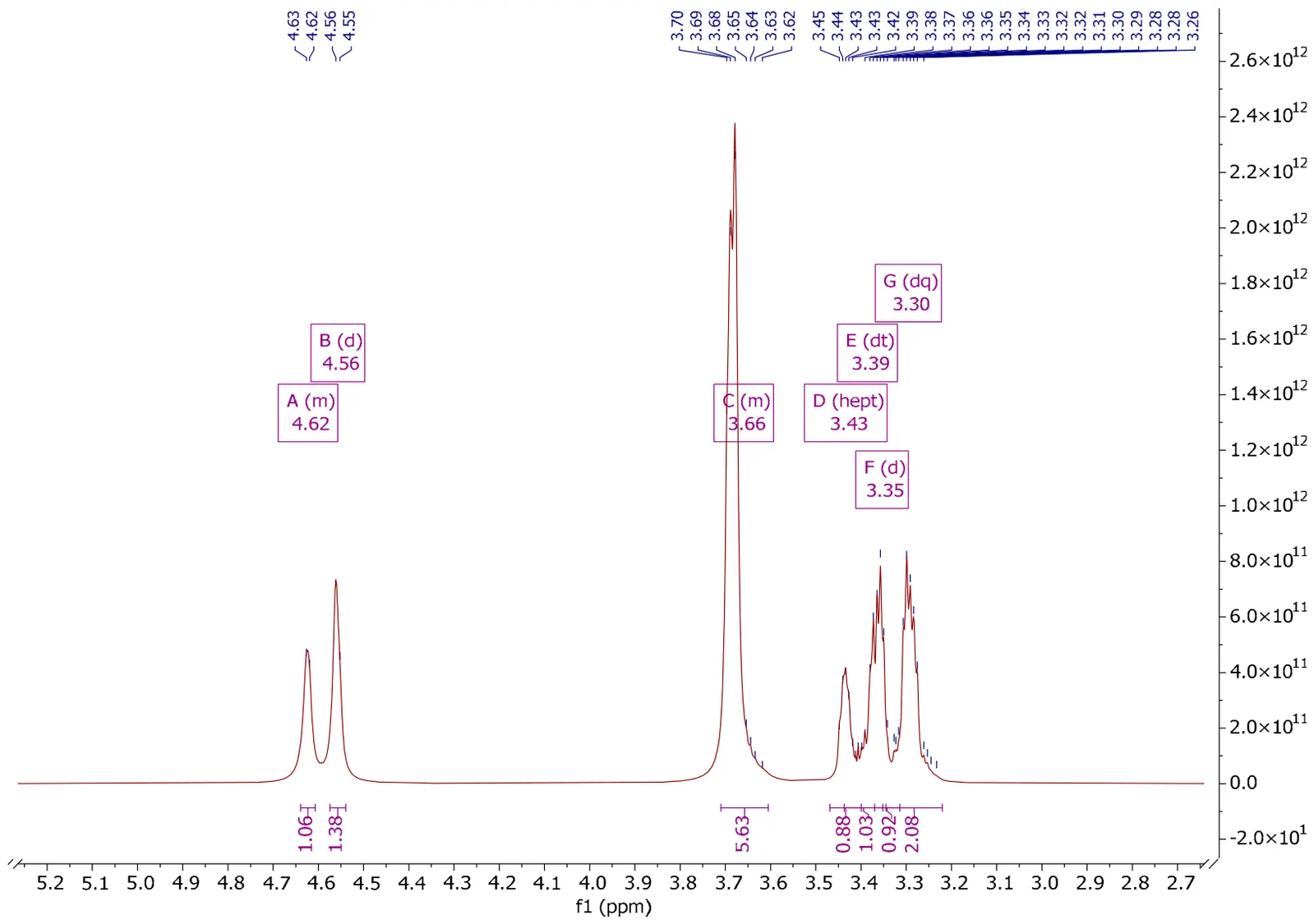
^1^H NMR spectrum of polyglycerol.

**Figure 8:**
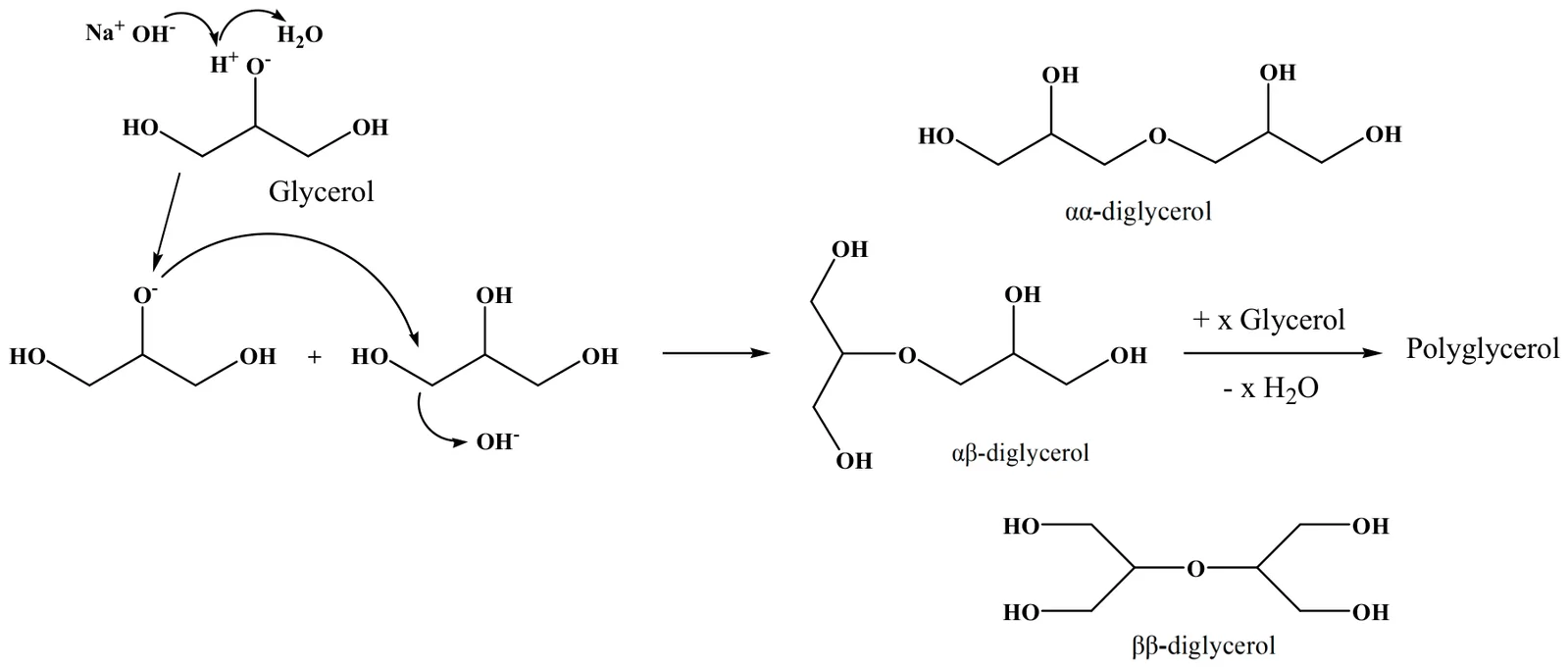
Mechanism of linear and branched glycerol polymerization reactions [16].

**Table 1:** Relationship between USG power and the resulting MW and DP in glycerol polymerization.

Sampel Code	Catalyst	Ampl	Output Power	Eergy	Viscosity	M^α^	Molecular Weight	Degree of Polymerization (DP)
		(%)	(Watt)	(Joule)	(η)		(gr/mol)	
GLYCEROL		0	0	0	0.499	25	92.78	1.01
HPG_N4_20	NaOH 4%	20	100	146,654	0.671	34	140.85	1.52
HPG_N4_30		30	150	268,521	1.189	59	315.32	3.41
HPG_N4_40		40	300	427,182	2.089	104	697.53	7.52
HPG_K4_20	KOH 4%	20	100	133,319	0.705	35	150.89	1.63
HPG_K4_30		30	150	255,958	1.057	53	267.16	2.88
HPG_K4_40		40	300	360,593	1.607	80	482.04	5.20

**Table 2:** FTIR spectral analysis table of polyglycerol.

Wavenumber (cm^−1^)	Functional Group	Vibration Mode
~3285.61	Hydroxyl (O–H)	Stretching
~2925.84–2873.17	Aliphatic (C–H)	Stretching (CH_2_)
~1655.88	Carbonyl (C=O) or C–O–C	Stretching
~1031.85	Ether (C–O–C)/Alcohol (C–O)	Stretching
1415.10–1100	C–H/CO	Bending/Stretching

**Table 3:** ^1^H-NMR peak assignments and structural unit quantification of the USG-synthesized hyperbranched Polyglycerol.

Structural Unit	Associated Peaks	Chemical Shift (δ, ppm)	Multiplicity	Integral Area
Dendritic (D)	A + B	4.62; 4.56	m; dt	1.06 + 1.38 = 2.44
Linear (L)	C	3.65	m	5.63
Terminal (T)	D + E + F + G	3.43; 3.39; 3.35; 3.30	Hepta; dt; d; quint	0.88 + 1.03 + 0.92 + 2.08 = 4.91

(m = multiplet, dt = doublet of triplets, hepta = heptalet, d = doublet, quint = quintet).

**Table 4:** Comparison of glycerol polymerization [4].

Catalyst	Temp. (°C)	Catalyst (wt%)	Time (h)	Reaction Conditions	Conversion (%)	Selectivity (PGx)	Reference
LiOH	240	2	6	Continuous flow, N_2_ atmosphere, Dean–Stark water removal	100	SPG2: 20%	[21]
LiOH	230–260	0.1	7	Low N_2_ flow, Dean–Stark water removal	24–80	SPG2–3: 63–100%	[22]
KOH/NaOH	260–280	2–4	1–4	Continuous N_2_ flow, removal of formed water	90–95	PG10–25	[23]
CsHCO_3_/Cs_2_CO_3_/CsOH	260	0.3–0.7	8	Atmospheric pressure	64–75	SPG2: 23–39%	[24]
Ca(OH)_2_	230	–	–	Vacuum (300 mmHg)	57	SPG2–3: 87%	[9]
Na_2_CO_3_/NaOH	240	0.1	9	–	63–76	SPG2–3: 93–99%	[25]
CsOH	260	2	4	N_2_ atmosphere, Dean–Stark water removal	90	SPG2–3: 63%	[26]
KOH/NaOH	260	–	–	Continuous N_2_ flow, removal of formed water	50–100	–	[8]
Ultrasound + NaOH	<100 (bulk)	4	2.5	Ultrasonic-assisted, no external heating, no inert gas	–	DB = 0.38; MW = 697.5 g/mol	This study

## Data Availability

The original contributions presented in this study are included in the article. Further inquiries can be directed to the corresponding author.
